# Supplementary material for: Dynamic Supercoiling Sponsors Transcription Amplification by MYC
Source: bioRxiv. 2026 Jan 19:2026.01.19.700398. Preprint. [Version 1] doi: 10.64898/2026.01.19.700398 (PMC12879662; doi:10.64898/2026.01.19.700398)
Supplement: Supplement 1 [file NIHPP2026.01.19.700398v1-supplement-1.pdf]

bioRxiv preprint doi: https://doi.org/10.64898/2026.01.19.700398; this version posted January 19, 2026. The copyright holder for this preprint (which was not certified by peer review) is the author/funder. This article is a US Government work. It is not subject to copyright under 17 USC 105 and is also made available for use under a CC0 license.

Supporting Information

Dynamic supercoiling sponsors transcription amplification by MYC

Rajiv Kumar Jha[1], Fedor Kouzine[1], Bo Wang[1], James D. Phelan[2], Subhendu K Das[1], Brian A Lewis[1],and David Levens[1]*

[1]Gene Regulation Section, Laboratory of Pathology, Center for Cancer Research, National Cancer Institute (NCI), Bethesda, MD 20892-1500, USA
[2] Lymphoid Malignancies Branch, National Cancer Institute, National Institutes of Health, Bethesda, MD 20892-1500, USA
*Corresponding author: levensd@mail.nih.gov

bioRxiv preprint doi: https://doi.org/10.64898/2026.01.19.700398; this version posted January 19, 2026. The copyright holder for this preprint (which was not certified by peer review) is the author/funder. This article is a US Government work. It is not subject to copyright under 17 USC 105 and is also made available for use under a CC0 license.

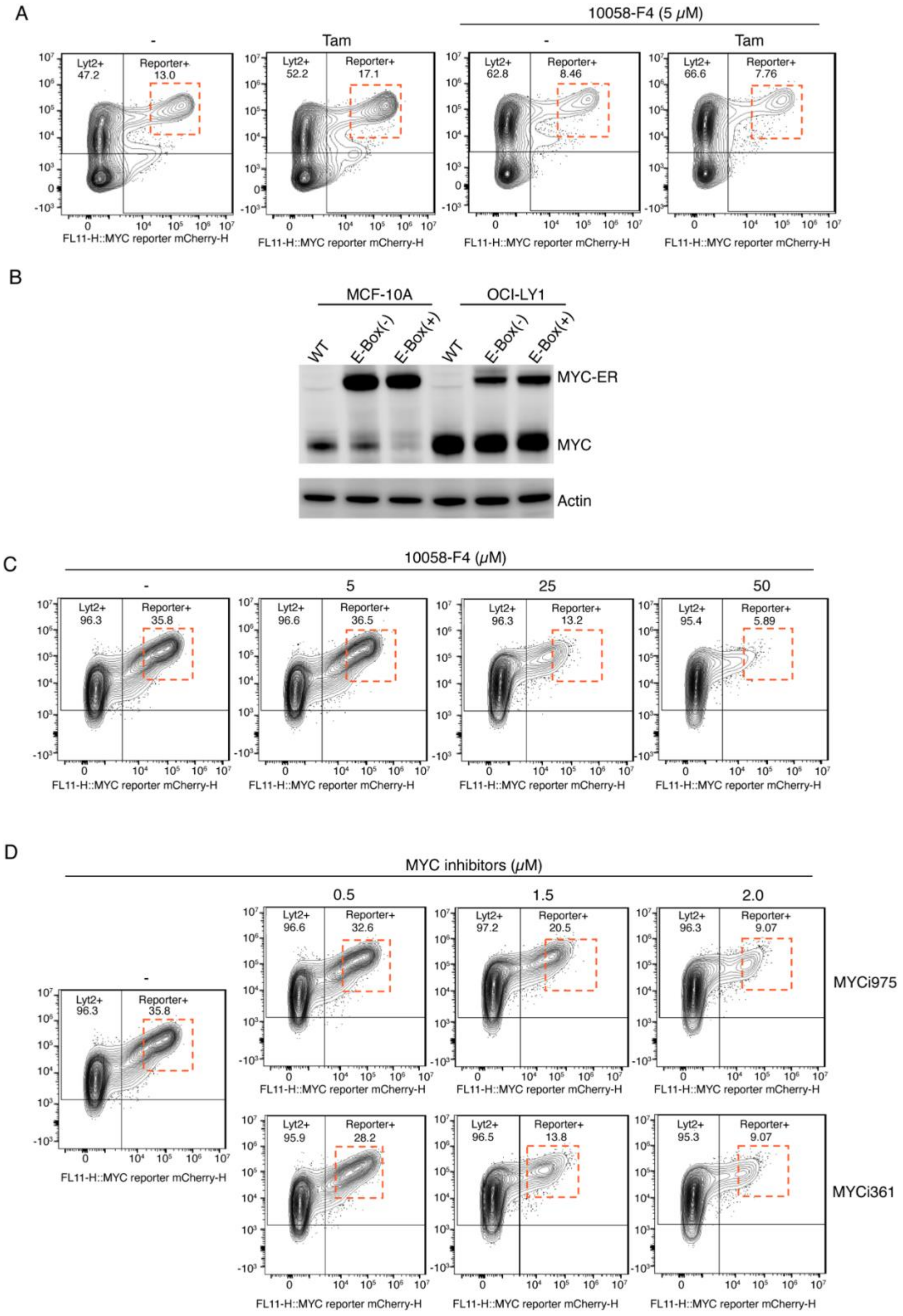

Figure S1

bioRxiv preprint doi: https://doi.org/10.64898/2026.01.19.700398; this version posted January 19, 2026. The copyright holder for this preprint (which was not certified by peer review) is the author/funder. This article is a US Government work. It is not subject to copyright under 17 USC 105 and is also made available for use under a CC0 license.

Fig. S1. A cell-based assay establishes MYC as a transcription amplifier. *(A)* MCF10A cells expressing lentiviral E-box+ mCherry constructs were cultured under indicated Dox/Tam conditions ± MYC inhibitor 10058-F4 (5 μM). Cells were analyzed by flow cytometry and mCherry fluorescence is shown on the X-axis, and LYT2 was detected using an anti-CD8α antibody on the Y-axis. Data were gated on live singlet cells gated for mCherry and LYT2 (Alexa-647) channels. Transcriptionally amplified populations are highlighted with red dashed box. *(B)* Immunoblot showing MYC and MYC-ER protein levels in MCF10A and OCI-LY1 cells treated with Dox (150 ng/ml) and Tam (200 μM). Actin served as a loading control. *(C)* OCI-LY1 lymphoma cells expressing lentiviral E-box+ mCherry constructs were under indicated Dox/Tam conditions ± MYC inhibitor 10058-F4 (5 -50 μM). Cells were analyzed by flow cytometry and mCherry fluorescence is shown on the X-axis, and LYT2 was detected using an anti-CD8α antibody on the Y-axis. Data were gated on live singlet cells gated for mCherry and LYT2 (Alexa-647) channels. Transcriptionally amplified populations are highlighted with red dashed box. *(D)* OCI-LY1 lymphoma cells expressing lentiviral E-box+ mCherry constructs were cultured under indicated Dox/Tam conditions ± MYC inhibitors MYCi975 or MYCi361 at various concentration (0.5 -2 μM). Cells were analyzed by flow cytometry and mCherry fluorescence is shown on the X-axis, and LYT2 was detected using an anti-CD8α antibody on the Y-axis. Data were gated on live singlet cells gated for mCherry and LYT2 (Alexa-647) channels. Transcriptionally amplified populations are highlighted with red dashed box.

bioRxiv preprint doi: https://doi.org/10.64898/2026.01.19.700398; this version posted January 19, 2026. The copyright holder for this preprint (which was not certified by peer review) is the author/funder. This article is a US Government work. It is not subject to copyright under 17 USC 105 and is also made available for use under a CC0 license.

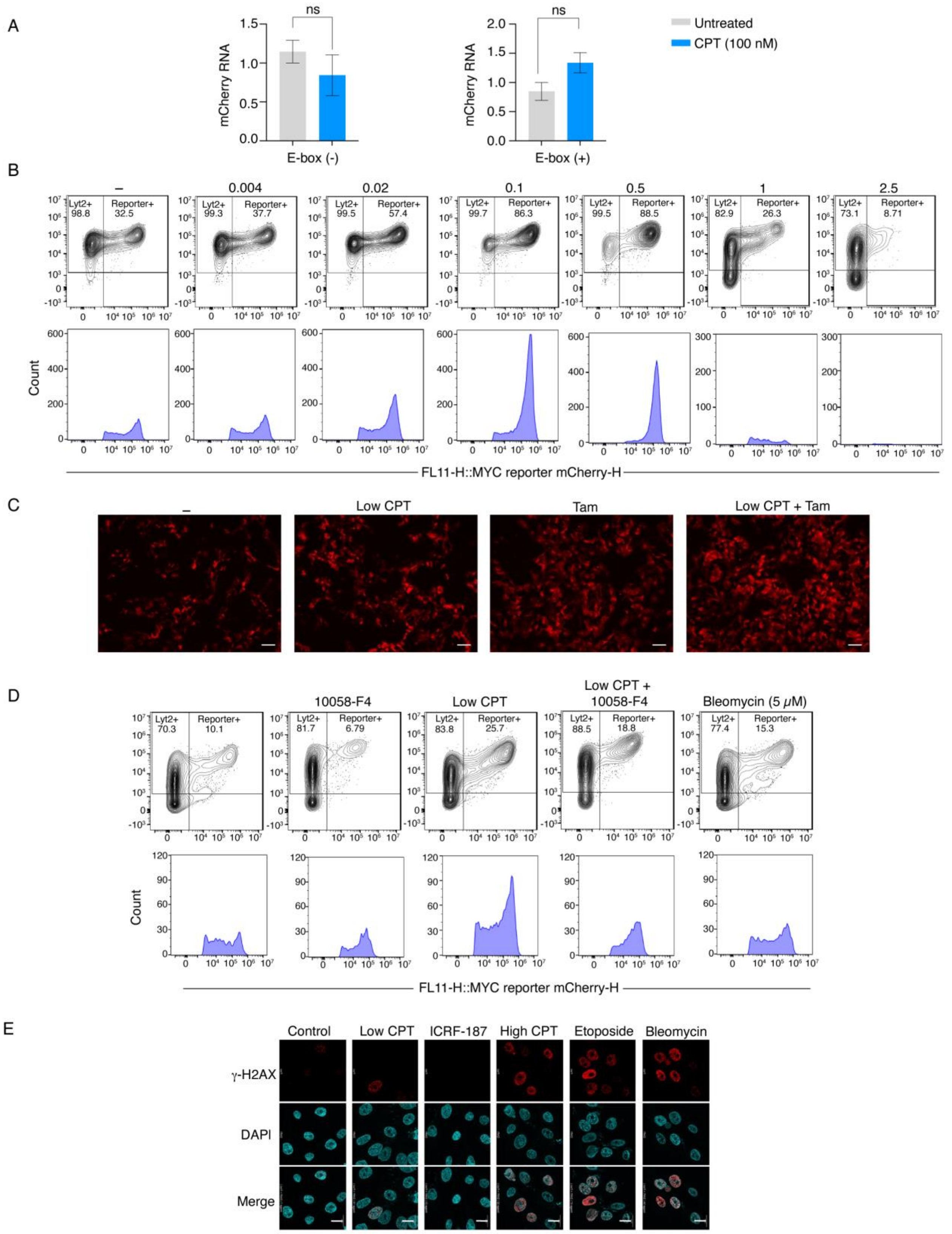

Figure S2

bioRxiv preprint doi: https://doi.org/10.64898/2026.01.19.700398; this version posted January 19, 2026. The copyright holder for this preprint (which was not certified by peer review) is the author/funder. This article is a US Government work. It is not subject to copyright under 17 USC 105 and is also made available for use under a CC0 license.

Fig. S2. Inhibition of TOP1 enhances transcription amplification by MYC, independent of DNA damage. *(A)* MCF10A cells expressing lentiviral mCherry constructs with or without E-box were treated with camptothecin (100 nM) and mCherry expression was quantified by RT-q-PCR (t-test, calculated values ± SEM from three biological replicates). *(B)* MCF10A cells expressing lentiviral E-box+ mCherry constructs were treated with Dox (150 ng/mL) ± Tam (200 μM) and treated with the indicated concentration of camptothecin. Cells were analyzed by flow cytometry and mCherry fluorescence is shown on the X-axis, and LYT2 was detected using an anti-CD8α antibody on the Y-axis. Data were gated on live singlet cells gated for mCherry and LYT2 (Alexa-647) channels. Histograms below show the fluorescence intensity distributions of mCherry-positive populations under each condition. *(C)* MCF10A cells expressing lentiviral E-box (–) mCherry constructs were cultured in the presence of Dox (150 ng/ml) in the presence or absence of Tam (200 μM) and CPT (100 nM). Representative microscopic images from at least three independent experiments are shown (scale bars, 200 μm). *(D)* MCF10A cells expressing lentiviral E-box (+) mCherry constructs were cultured with Dox (150 ng/mL) and treated with indicated combination of CPT (100 nM), 10058-F4 (5 μM), and bleomycin (5 μM). Cells were analyzed by flow cytometry and mCherry fluorescence is shown on the X-axis, and LYT2 was detected using an anti-CD8α antibody on the Y-axis. Data were gated on live singlet cells gated for mCherry and LYT2 (Alexa-647) channels. Histograms below show the fluorescence intensity distributions of mCherry-positive populations under each condition. *(E)* Immunofluorescence microscopy of MCF10A cells expressing lentiviral E-box (+) mCherry constructs under treatment with Dox (150 ng/mL) ± Tam (200 μM) and treated with the indicated topoisomerase inhibitors- low (100 nM) versus high dose CPT (2.5 μM), etoposide (20 μM), ICRF-187 (5 μM), and bleomycin (5 μM). γH2AX foci are red. Nuclei were stained with DAPI (scale bars, 25 μm).

bioRxiv preprint doi: https://doi.org/10.64898/2026.01.19.700398; this version posted January 19, 2026. The copyright holder for this preprint (which was not certified by peer review) is the author/funder. This article is a US Government work. It is not subject to copyright under 17 USC 105 and is also made available for use under a CC0 license.

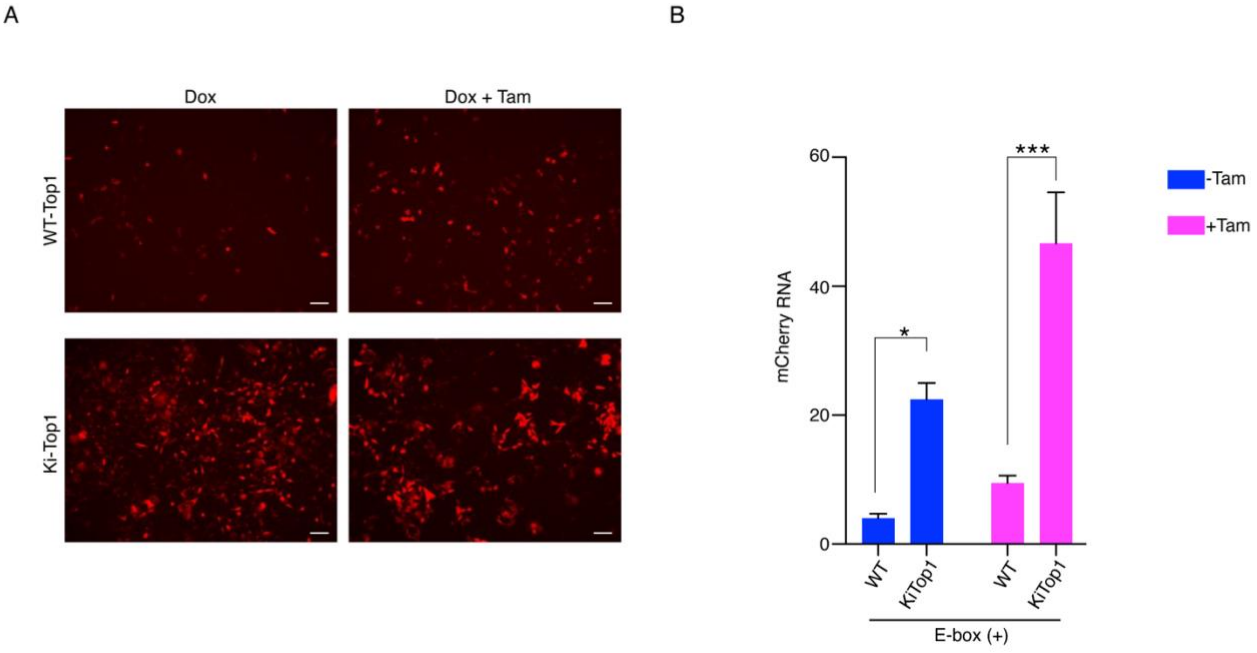

Figure S5

Fig. S5. DNA supercoiling pre-amplifies MYC-mediated transcription at promoters. *(A)* WT-TOP1 and KiTOP1 HCT116 cells expressing lentiviral E-box (+) mCherry constructs treated with the indicated conditions of Dox (150 ng/ml) and Tam (200 μM). Representative microscopic images from at least three independent experiments are shown (scale bars, 200 μm). *(B)* Quantification of fold change in mCherry mRNA expression in WT-TOP1 and KiTOP1 HCT116 cells expressing lentiviral E-box (+) mCherry constructs treated with the indicated conditions of Dox (150 ng/ml) and Tam (200 μM). Data represents mean ± SEM from three replicates. Asterisk denotes significant differences, for all statistical analyses (t-test): *, and ***, indicate $P < 0.05$,and 0.001, respectively.

bioRxiv preprint doi: https://doi.org/10.64898/2026.01.19.700398; this version posted January 19, 2026. The copyright holder for this preprint (which was not certified by peer review) is the author/funder. This article is a US Government work. It is not subject to copyright under 17 USC 105 and is also made available for use under a CC0 license.

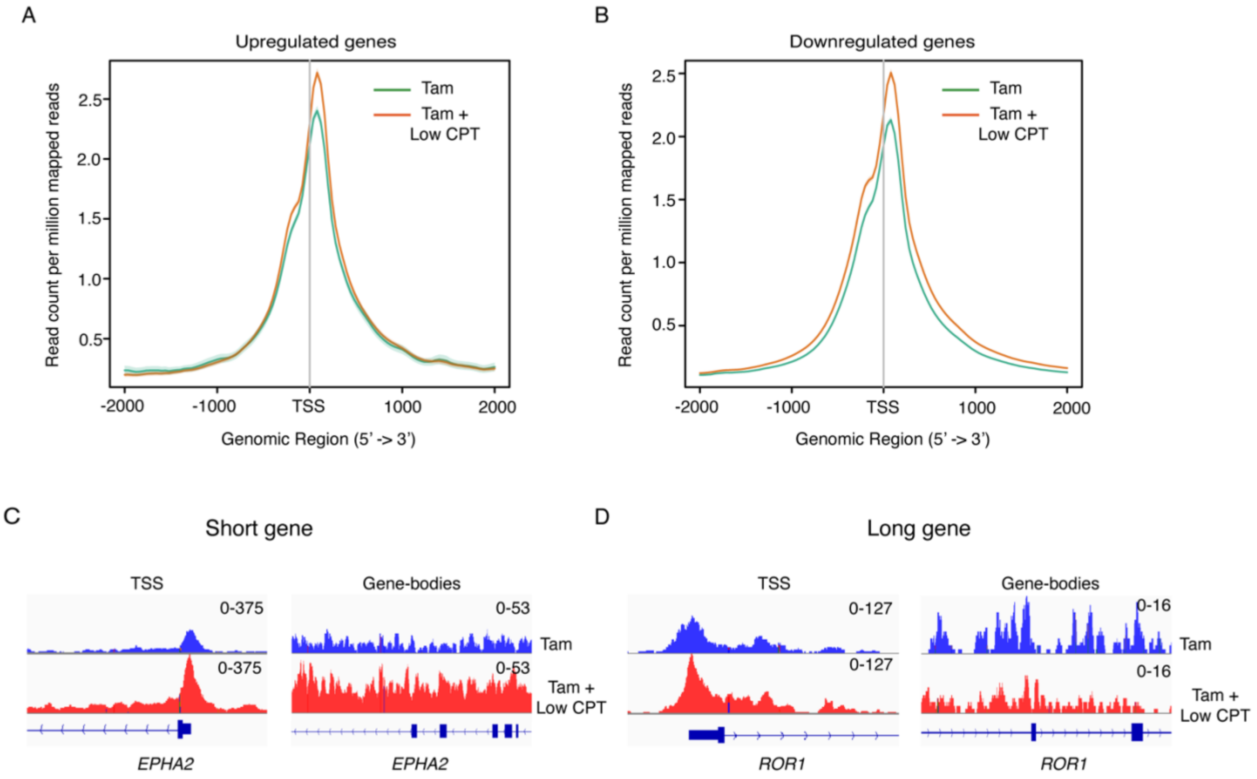

Figure S6

Fig. S6. Reduced TOP1 activity enhances RNAPII enrichment and facilitate transcription activation. *(A-B)* Metagene profiles of RNAPII occupancy (read counts per million mapped reads [RPM]) in MCF10A cells expressing lentiviral E-box (-) mCherry constructs after 24 h treatment with indicated combinations of Dox (150 ng/ml), Tam (200 µM) and CPT (100 nM). Metagene analyses of RNAPII occupancy (RPM) are shown at the TSS ± 2000 bp for upregulated genes (A), and downregulated genes (B) under the indicated conditions. (*C-D*) Genome browser tracks showing RNAPII occupancy at the short gene (*EPHA2*) and long gene (ROR1) locus. Signal intensities are displayed for the TSS region (left) and towards the TES (right). Blue traces represent the control [Tam], and red traces represent the Low CPT [Tam + LowCPT] condition.

bioRxiv preprint doi: https://doi.org/10.64898/2026.01.19.700398; this version posted January 19, 2026. The copyright holder for this preprint (which was not certified by peer review) is the author/funder. This article is a US Government work. It is not subject to copyright under 17 USC 105 and is also made available for use under a CC0 license.

Table S1: Cloning Primers

Primers	Sequence (5'-3')
mCherry-TetOne-FP	TTCCTACCACTTCCTACCCTCGTAAAGAATTCATGGTGAGCAAGGGCGAGGAGGATA
mCherry-TetOne-RP	TTAGCTCGCAGGGGAGGTGGTCTGGATCCTTACTTGTACAGCTCGTCCATGCCGCCGGTGGA
Ebox insertion-FP	ACGTGGTTTACATAAGCAAAGCTTATACG
Ebox insertion-RP	GGGCGCCTATAAAAGAGTGCTG
Plvx-AvrII-LYT2-FP	CCAGAAGTAGTGAGGAGGCTTTTTTGGAGGCCTAGGATGGCCTCACCGTTGACCCGC
Plvx-MluI-LYT2-RP	CCAGAGGTTGATTGTTCCAGACGCGTCTAGCGGCTGTGGTAGCAGATGAG
WPRE-sNRP1polyA-FP	ATAAAATACGAAATGTGACAGAATTAATTCTGCAGTCGAGACCTAG
WPRE-sNRP1polyA-RP	TTGATACCTGATTGTATGGTCAGGCGGGGAGGCGGCCCAAAG
EF1A-kpn1-FP	CAGTCGAGACCTAGAAAAACATGGTACCGCTCCGGTGCCCGTCAGTGGGC
EF1A-AgeI-RP	CGTTGAGGGGCATATTTAAATACCGGTGGTGGGCGCGCCAAGCTTCCACG
MycER-PolyA-TetOne-FP	GAGGAGGTGGGTTTTTCCAGTCACACCAGCTAGCTGCAGTAACGCCATTTTG
MycER-PolyA-TetOne-RP	GTAAGTCATTGGTCTTAAAGGTACCTGAGATCCAGACATGATAAGATACATTG
SFFV-del-FP	TACCGACAGACTGAGTCGGCCG
SFFV-del-RP	CCATGTTTTTCTAGGTCTCGACTGCAG
EF1A-Kpn1-MycER-FP	GCAGTCGAGACCTAGAAAAACATGGTACCGCTCCGGTGCCCGTCAGTGGGC
EF1A-AgeI-MycER-RP	CGTTGAGGGGCATATTTAAATACCGGTGGTGGGCGCGCCAAGCTTCCACG

Table S2: Real-time PCR and qPCR primers

Primers	Sequence (5'-3')
mCherry-gene-RT-FP	GAGATCAAGCAGAGGCTGAA
mCherry-gene-RT-RP	CCAACTTGATGTTGACGTTGTAG
mCherry-Promoter-RT-FP	TCCATGTGCACCTTGAAGC
mCherry-Promoter-RT-RP	TGAGTAAACTTCAATTCCACAACAC
mCherry-Promoter-qPCR-FP	TGATAATTCCACGGGGTTGG
mCherry-Promoter-qPCR--RP	TGCGAACGGACGTGAAGAAT
Actin-FP	CACCATTGGCAATGAGCGGTTC
Actin-RP	AGGTCTTTGCGGATGTCCACGT

bioRxiv preprint doi: https://doi.org/10.64898/2026.01.19.700398; this version posted January 19, 2026. The copyright holder for this preprint (which was not certified by peer review) is the author/funder. This article is a US Government work. It is not subject to copyright under 17 USC 105 and is also made available for use under a CC0 license.

Table S3: Reagents and Source

REAGENT or RESOURCE	SOURCE	IDENTIFIER
**Chemicals**		
DMEM, high glucose	Thermo Fisher Scientific	Cat# 11965092
Opti-MEM™ I Reduced Serum Medium	Thermo Fisher Scientific	Cat# 31985062
Corning® 100 mL MEM Nonessential Amino Acids, 100x	Corning	Cat# 25-025-CI
Penicillin-Streptomycin (10,000 U/mL)	Gibco	Cat# 15140122
Bovine Serum, heat inactivated, New Zealand origin	Thermo Fisher Scientific	Cat# 26170043
Charcoal/Dextran Stripped Fetal Bovine Serum	GeminiBio	Cat# 100119500
DMEM/F-12	Invitrogen	Cat# 11330032
Horse Serum, New Zealand origin	gibco	Cat# 16050114
EGF	Peprotech	Cat# AF-100-15
Hydrocortisone	Milipore-Sigma	Cat# H0888
Cholera Toxin	Milipore-Sigma	Cat# C8052
Insulin	Milipore-Sigma	Cat# I1882
DharmaFECT™ 2 Transfection Reagent	Horizon	Cat# T-2002-03
ON-TARGETplus Smartpool Human TOP1 siRNA	Horizon	Cat# L-005278-00-0005
ON-TARGETplus Smartpool Human TOP2A siRNA	Horizon	Cat# L-004239-00-0005
Non-targeting control siRNA	Horizon	Cat# D-001210-02-05
cOmplete™, Mini, EDTA-free Protease Inhibitor Cocktail	Milipore-Sigma	Cat# 11836170001
Tamoxifen	Milipore-Sigma	Cat# T5648-1G
ICRF-187	Selleck Chemicals LLC	Cat# S1222
Etoposide	Milipore-Sigma	Cat# E1383-250MG
Bleomycin	Milipore-Sigma	Cat# BP971
Camptothecin	Milipore-Sigma	Cat# C9911-250MG
UltraPure™ Agarose	Thermo Fisher Scientific	Cat# 16500500
VECTASHIELD® Antifade Mounting Medium with DAPI	Vector laboratories	Cat# H-1200-10
Pierce™ Protein A/G Magnetic Beads	Thermo Fisher Scientific	Cat# 88803
RNase A, DNase and protease-free (10 mg/mL)	Thermo Fisher Scientific	Cat# EN0531

bioRxiv preprint doi: https://doi.org/10.64898/2026.01.19.700398; this version posted January 19, 2026. The copyright holder for this preprint (which was not certified by peer review) is the author/funder. This article is a US Government work. It is not subject to copyright under 17 USC 105 and is also made available for use under a CC0 license.

Sodium deoxycholate monohydrate BioXtra, ≥99.0% (titration)	Milipore-Sigma	Cat# D5670-25G
Urea ACS reagent, 99.0-100.5%	Milipore-Sigma	Cat# U5128-5KG
DTT 1,4-Dithiothreitol	Milipore-Sigma	Cat# 11583786001
Nonidet™ P 40 Substitute	Milipore-Sigma	Cat# 74385
Ethylenediaminetetraacetic acid disodium salt dihydrate	Milipore-Sigma	Cat# 03685-500G
Exonuclease I	New England Biolabs Inc.	Cat# M0293L
NuPAGE™ LDS Sample Buffer (4X)	Thermo Fisher Scientific	Cat# NP0007
Pierce™ 16% Formaldehyde (w/v), Methanol-free	Thermo Fisher Scientific	Cat# 28906
4-12% Bis-Tris NuPAGE protein gel	Thermo Fisher Scientific	Cat# NP0323
SuperSignal™ West Femto Maximum Sensitivity Substrate	Thermo Fisher Scientific	Cat# 34095
Nitrocellulose Membranes, 0.45 µm	Thermo Fisher Scientific	Cat# 77010
Sodium Chloride	Milipore-Sigma	Cat# S9888
Bovine Serum Albumin	Milipore-Sigma	Cat# A7906
Ethyl Alcohol	Milipore-Sigma	Cat# E7023
Magnesium chloride	Milipore-Sigma	Cat# M8266
Direct-zol RNA Miniprep Kits	Zymo-Research	Cat# R2053
ChIP-IT High Sensitivity	Activ Motif	Cat# 53040
SYBR™ Green I Nucleic Acid Gel Stain	Thermo Fisher Scientific	Cat# S7563
Phenol:Chloroform:Isoamyl Alcohol 25:24:1 Saturated with 10 mM Tris, pH 8.0, 1 mM EDTA	Milipore-Sigma	Cat# P2069-400ML
Proteinase K, Molecular Biology Grade	New England Biolabs Inc.	Cat# P8107S
4,5′,8- trimethylpsoralen	MP Biomedicals	Cat# 154157
32-CTP 3000 Ci/mmol, 0.25 mCi	Perkin Elmer Lifer Sciences	Cat# BLU008H250UC
**Antibodies**		
Rabbit monoclonal Anti-Topoisomerase I antibody	Abcam	Cat# ab109374; RRID:AB_10861978
Rabbit monoclonal Recombinant Anti-c-Myc antibody [Y69]	Abcam	Cat# ab32072; RRID:AB_731658
RNA pol II antibody	Active Motif	Cat# 102660 RRID:AB_2687451
normal rabbit IgG antibody	Santa Cruz Biotechnology	Cat# sc-2027; RRID:AB_737197

bioRxiv preprint doi: https://doi.org/10.64898/2026.01.19.700398; this version posted January 19, 2026. The copyright holder for this preprint (which was not certified by peer review) is the author/funder. This article is a US Government work. It is not subject to copyright under 17 USC 105 and is also made available for use under a CC0 license.

Goat Anti-Rabbit IgG H&L (HRP) antibody	Abcam	Cat# ab205718; RRID:AB_2819160
mouse monoclonal anti-actin	Cell Signaling Technology	Cat# 3700; RRID:AB_2242334
Alexa FluoR^{R} 647 anti-mouse CD8a	BioLegend	Cat# 100724 RRID:AB_389326
Rabbit monoclonal Anti-Topoisomerase II alpha antibody	Abcam	Cat# ab52934; RRID:AB_883143
